# Which green way: description of the intervention for mobilising against *Aedes aegypti* under difficult security conditions in southern Mexico

**DOI:** 10.1186/s12889-017-4300-1

**Published:** 2017-05-30

**Authors:** Arcadio Morales-Perez, Elizabeth Nava-Aguilera, José Legorreta-Soberanis, Sergio Paredes-Solís, Alejandro Balanzar-Martínez, Felipe René Serrano-de los Santos, Claudia Erika Ríos-Rivera, Jaime García-Leyva, Robert J. Ledogar, Anne Cockcroft, Neil Andersson

**Affiliations:** 1 Centro de Investigación de Enfermedades Tropicales (CIET), Universidad Autónoma de Guerrero, Acapulco, Mexico; 2 CIETinternational, New York, NY USA; 3 Department of Family Medicine, McGill University, Montreal, Canada; 4 CIET Trust, Gaborone, Botswana

**Keywords:** Dengue vector control, Camino Verde, Community mobilisation, Mexico, Implementation

## Abstract

**Background:**

Community mobilisation for prevention requires engagement with and buy in from those communities. In the Mexico state of Guerrero, unprecedented social violence related to the narcotics trade has eroded most community structures. A recent randomised controlled trial in 90 coastal communities achieved sufficient mobilisation to reduce conventional vector density indicators, self-reported dengue illness and serologically proved dengue virus infection.

**Methods:**

The *Camino Verde* intervention was a participatory research protocol promoting local discussion of baseline evidence and co-design of vector control solutions. Training of facilitators emphasised community authorship rather than trying to convince communities to do specific activities. Several discussion groups in each intervention community generated a loose and evolving prevention plan. Facilitators trained *brigadistas*, the first wave of whom received a small monthly stipend. Increasing numbers of volunteers joined the effort without pay. All communities opted to work with schoolchildren and for house-to-house visits by *brigadístas*. Children joined the neighbourhood vector control movements where security conditions permitted. After 6 months, a peer evaluation involved *brigadista* visits between intervention communities to review and to share progress.

**Discussion:**

Although most communities had no active social institutions at the outset, local action planning using survey data provided a starting point for community authorship. Well-known in their own communities, *brigadistas* faced little security risk compared with the facilitators who visited the communities, or with governmental programmes. We believe the training focus on evidence-based dialogue and a plural community ownership through multiple design groups were key to success under challenging security conditions.

**Trial registration:**

ISRCTN27581154.

## Background

With zika and chikungunya on the rise alongside dengue, control of the *Aedes aegypti* mosquito has probably never been such an international priority as it is now. For the last 20 years or more, government programmes to contain the mosquito have relied on chemical control using temephos. Over the last 20 years, the steady increase of *Aedes*-related diseases alongside ever increasing disbursements of pesticide suggest these chemical approaches have been futile; there is also accumulating evidence of resistance to temephos [1, 2].

The *Camino Verde* cluster randomised controlled trial (ISRCTN27581154) tested the impact of chemical-free evidence-based community mobilisation in Managua in Nicaragua, and in the southern Mexican state of Guerrero. Earlier trials of community-based activities had reported an impact on entomological indices of the *Aedes* vector [3, 4], but *Camino Verde* was the first trial to demonstrate an impact on serological evidence of dengue virus infection. The intervention was not a fixed set of vector control activities but a protocol for communities themselves to identify preventive actions after discussion of baseline evidence of vector reproduction sites [5]. Primary outcomes *per protocol* were serological evidence of dengue virus infection (children aged 3–9 years) in paired saliva samples collected before and after the dengue season, self-reported dengue cases in the last 12 months, and conventional entomological indices after 18 months of intervention. With cluster as the unit of analysis, after one dengue season and compared with 75 control sites, 75 intervention sites showed lower risk of dengue virus infection in children (RRR 29.5%, 95% CI 3.8 to 55.3), dengue illness (RRR 24.7%, 95% CI 1.8 to 51.2), house index (RRR 44.1%, 95% CI 13.6 to 74.7), container index (RRR 36.7%, 95% CI 24.5 to 44.8), Breteau index (RRR 35.1% 95% CI 16.7 to 55.5) and pupae per person (RRR 51.7%, 95% CI 36.2 to 76.1).

Community mobilisation requires engagement and buy in. There are well recognised challenges in successful mobilisation, and inappropriate assumptions about shared interests of public services and residents [6, 7] and ignorance about the cost or work implications for residents [8]. Added to these challenges in Mexico, social violence generated by the narcotics trade has eroded if not eliminated most local community structures [9–11]. Since 2012, Acapulco has been one of the four most dangerous cities in the world [12] and, according to the Institute for Economy and Peace, in 2015 Guerrero eclipsed even Sinaloa and Chihuahua, infamous for their heroin trade violence, to become Mexico’s most violent state [13]. How *Camino Verde* was implemented and achieved an impact in the face of these daunting levels of violence might be informative and encouraging to those interested in chemical-free containment of *Aedes aegypti* in other settings.

## Methods

### Socialising evidence for participatory action (SEPA)

We reported the *Camino Verde* trial methods elsewhere [5]. In its Mexican arm, the trial allocated 90 clusters, each of about 140 households, to intervention (45 clusters) or control (45 clusters) status after a baseline household survey. Both arms continued the usual government vector control programme, including temephos deposits in household water containers and occasional space fumigation by trucks driving through the community and aerial spraying from low flying aircraft. The intervention was a four-point protocol that included stakeholder discussion of baseline evidence, co-design of prevention activities, training of community volunteers, and peer evaluation for inter-community sharing and learning [14]. The central dynamic was Socialising Evidence for Participatory Action (SEPA) [15, 16], sharing and discussing evidence in a way that supports stakeholders to decide upon and take actions that change their own situation. SEPA was inspired by conscientizing concepts introduced in Latin America by Paulo Freire [17, 18] and ownership of research tools and products by the Italian labour movement’s *alternative operaia* or workers’ model [19]*.* Run by workers themselves, this model documents the daily work experience in the context of the labour process, to inform analysis of work related accidents and morbidity. An article by Ledogar and colleagues describes SEPA in more detail [20].

The following sections describe the intervention teams in the Mexican arm of the trial, their engagement with communities to share information and to plan prevention activities, and activities at household and community levels, including interactions with service providers.

### Who did the work?

#### The researchers

The *Centro de Investigacion de Enfermedades Tropicales* (CIET), an academic unit within the *Universidad Autónoma de Guerrero* (UAGro), is widely known and respected in Guerrero for its 30 years of community-based research. CIET researchers trained the facilitators.

#### Facilitators

Recruited among recent psychology or social anthropology graduates from the UAGro to work as intermediaries between the research team and the community volunteers or *brigadistas,* facilitators provided training and support. Some 30 short-listed candidates attended training by a CIET team from Mexico and Nicaragua, and a Mexican social mobilisation expert. The classroom training over 2 weeks covered the philosophy and approach of community-based research, the trial design, results from the baseline survey, the life cycle of *Aedes aegypti* and related control mechanisms, symptoms of dengue illness, focus group facilitation and approaches to communication. Over a further month of practical training in Acapulco, facilitators learned how to conduct entomological inspections of household water containers and to work with community members in groups. The CIET team selected 15 facilitators from the 30 who attended the training. After their training, each facilitator assumed responsibility for three intervention sites in the three coastal regions.

#### Brigadistas

The team recruited four *brigadistas* from each the 45 intervention communities. CIET researchers accompanied the facilitators to their assigned communities and presented them to local authorities and, where these existed, neighbourhood leaders, who then called community meetings to provide information about the project. Especially in rural sites, *brigadistas* often came forward during these meetings. In other sites, the CIET researchers and facilitators contacted people suggested by community leaders. In a few communities, the researchers recruited the *brigadistas* among those who had conducted the baseline household survey of the trial. Most urban sites had poorly defined local leadership structures. Here members of the parents’ committees in the schools, or itinerant vendors who knew the communities well helped to identify people who might be approached to be *brigadistas*. All *brigadistas* were members of the communities in which they worked, and were known to the residents. Based on the prominent role of women in household maintenance and child care, and in the knowledge that the intervention was likely to be quite empowering, the researchers encouraged women to join; around 70% of recruits were female. Selection of a younger and an older person in each pair of *brigadistas* resulted in a bimodal age distribution with peaks in the early 20s and around 50 years.

Facilitators trained the *brigadistas* over 1 week before visiting households, and accompanied them for a further month in the field. *Brigadista* training and supervision emphasised respect for householders irrespective of age, educational level, religion and customs. The training covered informed consent for entering households to inspect the water containers; confidentiality; and willingness to act on householder suggestions for dengue control efforts. *Brigadistas* learned about the life cycle of the *Aedes aegypti* mosquito by adding mosquito eggs to clean water, in a jar or plastic bag, and observing the daily development from egg, to larva, to pupa, to adult mosquito. The first training covered the practical task of returning results from the baseline saliva samples to households that contributed samples.

### Engaging with communities to plan activities

Facilitators and *brigadistas* organized several discussion groups in each of 45 intervention clusters. Linkages between the project and existing social infrastructure were prominent in the Nicaraguan arm of the trial, where involvement of existing groups was pivotal to the *Camino Verde* intervention in that country [21]. But the situation was very different in Guerrero; key informants interviewed in the baseline survey identified the existence of *any* organised community groups in only 11 of the 45 intervention clusters and 10 of the 45 control clusters [22]. Three of every four communities (69/90) had *no* active social institutions or groups at baseline.

In communities with active social groups, these were a natural starting point for SEPA. In others, facilitators tried to engage organisational remnants including school parents’ associations, religious organisations, Alcoholics Anonymous groups, sports groups, marketers, and groups established by the government *Oportunidades* programme (that provided cash incentives for families that agreed to participate in health, nutrition and educational activities). Some 43 of the groups identified were all male, 45 of them all female and 47 were mixed groups of professionals like teachers and health workers; another 47 groups were children’s groups. Adult groups had a wide spread of ages, between 20 and 60 years or more.

The discussion groups had three main purposes: to share information obtained in the baseline survey and about the mosquito life cycle; to identify actions at household level to control the mosquito; and to identify dissemination strategies that *brigadista*s could use in the community. Details of the presentation of evidence and processes for stimulating deliberative dialogue are the subject of an article by Ledogar and colleagues [20]. As part of their input, facilitators provided information on illustrative activities from the Managua feasibility study, some of which resonated in the Guerrero context.

### Locally-defined vector control actions at household level

**Fig. 1:**
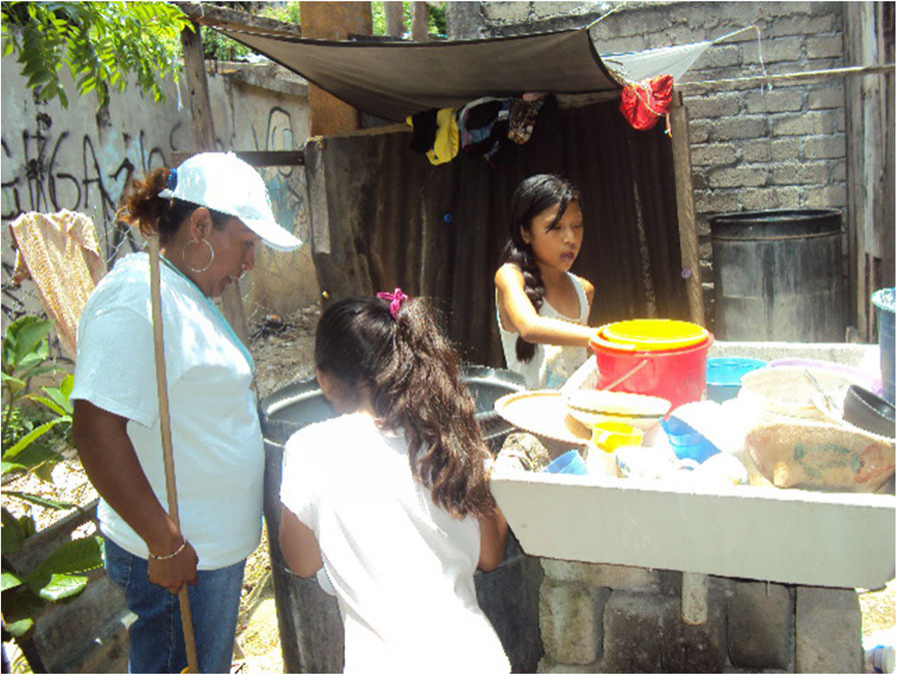
A brigadista examines water containers with children during a household visit

After the baseline study, the *brigadistas* returned results of the saliva sample tests. They approached every family in both intervention and control communities whose child provided two saliva samples during the baseline survey. In all communities they referred all families whose child showed evidence of recent dengue infection to the appropriate health services. In the intervention communities they also spent time introducing and discussing the project to prevent dengue. This probably spurred the proposal by all community planning groups that *brigadistas* should do regular household visits (*visitas de acompañamiento*). During these visits to all households the *brigadistas* shared evidence about the life cycle of the dengue vector and demonstrated inspection of water containers on the premises that were potential breeding places for mosquitoes (Fig. 1). More a dialogic than a supervisory activity, the household visits were intended to fuel discussions about prevention.

Tools the *brigadistas* used during their visits included a laminated card with a diagram of the mosquito’s life-cycle, a water jar or plastic bag containing live mosquito larvae and simple aids (a strainer, flashlight and pipette) for collecting larvae. They invited the householders to watch development of the larvae into mature mosquitoes. Household visits were not structured by protocol but all started by reviewing breeding sites and identifying eggs, larvae and pupae, going on to discuss and to demonstrate how to deal with them. Once households were familiar with these procedures, visits concentrated on reviewing the state of breeding sites and discussing other possible sites, for example in vacant dwellings.

In the planning of the trial, we scheduled one visit per week to each household. As householders got to know the *brigadistas,* these visits increased, with *brigadistas* spending about 14 h per week on this activity. Each pair of *brigadistas* started by visiting a few households in a day, building up to 15–20 households in a day as their time in each household shortened. They visited all households included in each self-defined community; visits were not limited to the households surveyed. Each household was probably visited about 20 times over the 3 months of the main dengue season.

The initial group of four *brigadistas* per community received a stipend slightly less than the local cost of one meal for each day worked – around USD90 per month. This undoubtedly contributed to compliance and accountability. The decision by the research team to pay the initial cohort was based on early recruitment difficulties. As the process gained momentum, particularly as a large number children became involved, there was neither the need to pay nor, given the fixed project budget, the ability to do so.

### Locally-defined vector control actions at community level

**Fig. 2:**
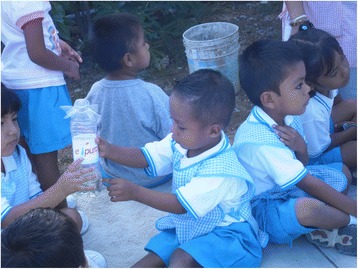
Even young children became fascinated by the stages of mosquito development

**Fig. 3:**
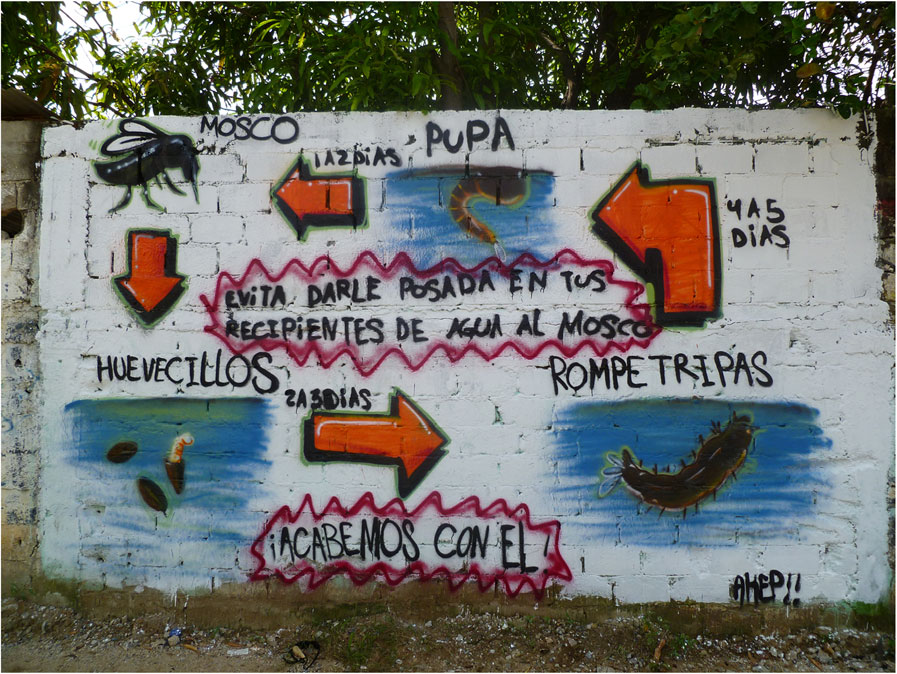
A mural of the *Aedes aegypti * life cycle

**Fig. 4:**
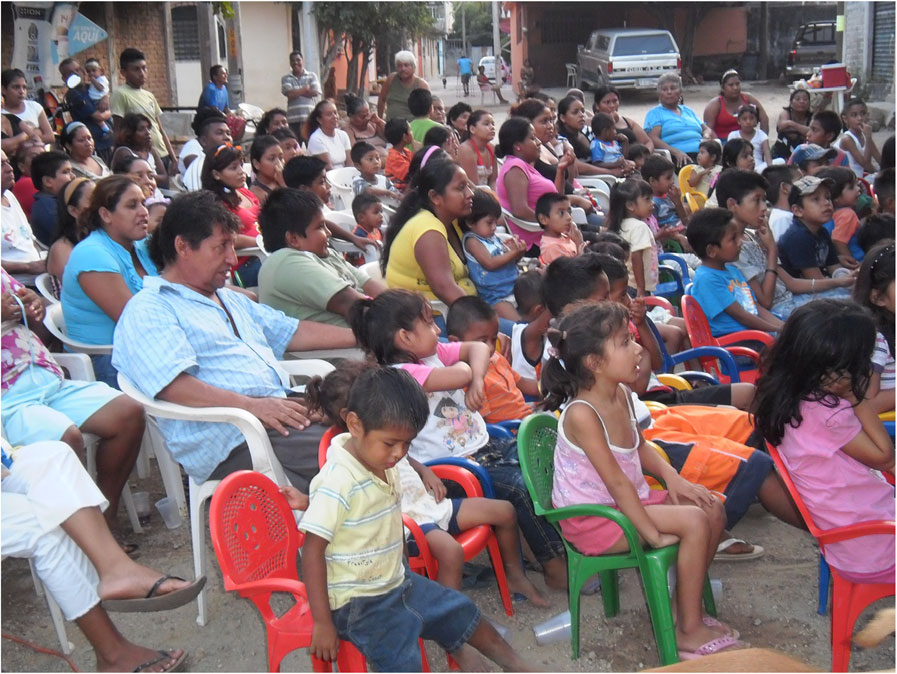
The audience enjoying a drama about the dengue vector

In every intervention community, action planning groups suggested working with the local schools. *Brigadistas* and their facilitator met the director and teachers of each school to discuss the evidence from the baseline survey and to select student *brigadistas* to lead activities at the school. The facilitators and *brigadistas* then worked with teachers to develop ways to raise dengue awareness in their schools (Fig. 2). Lotteries, word games, piñatas, kites and pencils decorated with images of the stages of mosquito development became ways of sharing the evidence about dengue with the children. Socio-dramas, plays and puppet shows were all ways for sharing dengue prevention content (Figs. 3 and 4). The schools turned out to be centres of efforts to control the dengue vector in most communities, often leading to activities outside the schools such as sports competitions and local marches about the dengue vector.

As activities of the intervention became more visible, more community members joined in. *Brigadistas* in each community also interacted with local service providers. With local health services, as a locally defined initiative, they synchronised educational messages about dengue prevention and treatment, and referred cases of suspected dengue to the services. Also as local initiatives, most community teams liaised with the local Social Development Secretariat for their area to share evidence about dengue prevention with beneficiaries of the government programme *Oportunidades*. Supported by community members, *brigadistas* sought the cooperation of the Water and Sanitation Departments in community clean-up activities.

#### Biological control using fish

Using fish for biological control of *Aedes aegypti* breeding was not part of the training of facilitators. Planning groups in several rural intervention communities reported that, prior to the government programme of chemical control with temephos, they placed small fish in their water containers as these ate the mosquito larvae. Community members were keen to return to this practice that had been eliminated with the use of temephos. *Brigadistas* helped residents to catch suitable fish from local ponds and streams, then showed other residents how these fish eat larvae. Many householders began replacing temephos sachets with biological control. The practice spread through word of mouth between *brigadista*s to other communities, particularly in the rural coastal regions. Some communities adopted larger fish like tilapia, and crustaceans like crayfish. By the time of the trial impact survey at the end of 2012, one in every four households in intervention communities kept fish in their water storage containers [23].

### The mid-course peer evaluation

After about 6 months of fieldwork, *brigadistas* from each site visited another intervention community. They applied a brief questionnaire and conducted joint entomological inspections in consenting households. The visiting *brigadistas* presented their findings to the host *brigadistas* and together they discussed possible adjustments to the approaches being used. In these exchanges, *brigadistas* shared successes and challenges, learned about innovative prevention activities, and documented what participants in the other sites felt had worked best for them.

### Implementation challenges

Security concerns in most of the communities meant people were initially unwilling to attend meetings; attention to venue, often at schools over the weekend, and timing, during daylight hours, helped to overcome this problem.

The lack of organised social activities in most communities presented a challenge to convene action planning groups and all steps after that. *Brigadistas* used their personal contacts to convene enough residents who had knowledge of the community and who could contribute to developing strategies for reaching and informing other community members about the trial.

Generating enthusiasm for the intervention was difficult because few people saw dengue as an actionable priority problem. Decades of governmental use of temephos and aerial spraying had led them to believe this was not something they could control. Conflicts among neighbours, scarcity of water supply, lack of services, insecurity, land invasions by squatters and other health problems were of more immediate concern. The facilitators and *brigadista*s had to work hard to convince people that dengue was infecting many of their children, that dengue cases could be a financial burden on households, and that they themselves could do something about it.

At the start of the intervention, most local health authorities were not interested; they considered dengue prevention to be a government responsibility to be solved with the government temephos and spraying programmes. As the *brigadistas* shared the evidence about non-chemical ways to control the vector within their communities, confidence in this approach grew. Attitudes among local health authorities began to change and they started to support *brigadistas* in their tasks and even to join in community efforts without asking for anything in exchange. This in turn won health care providers more respect in the communities.

Incorporation of different age groups in the brigades proved successful. We initially thought children would work only with their peers but they gradually became part of the general neighbourhood movements. Children were good at socialising the evidence among their peers, and helpful to the teams in gaining entry to households. Their enthusiasm for discovering mosquito larvae in water containers motivated adults to join in the effort.

*Brigadistas* working within their own communities and well-known in those communities faced relatively little risk. But the facilitators did not live in the communities where they worked and had to use public transport to reach them, sometimes covering considerable distances. The project provided insurance to cover possible accidents and associated medical costs, fortunately not required during the trial. Safety rules included the facilitators checking security conditions with the *brigadista*s before travelling to communities. All field team members wore identifying t-shirts and caps bearing the Camino Verde logo, and were forbidden to wear dark glasses (emblematic of those involved in drug trafficking) or expensive jewellery, to carry expensive cell phones, or to consume alcohol while working in the communities.

## Discussion and conclusions

We believe the success of *Camino Verde* is better explained by the participatory engagement process than it is by the specific actions they chose or the particularity of the research organisation or researchers – very little of the fieldwork and none of the actual community mobilisation was done by CIET researchers.

SEPA has its origins in Latin American education theory rather than in communication theory and in the *Camino Verde* trial was concerned more with the process of community authorship and ownership than specific predetermined activities; communities chose different combinations of actions.

The first and probably most important factor contributing to this community ownership was the quite detailed facilitator training, including a month of accompanied work before final selection of facilitators. Trainees selected to work as facilitators were those who could best facilitate discussions, rather than those who could “lead” the community. There was never any sense of facilitators “getting communities to do what we think should be done”.

Another factor was the diversity of community involvement. No less than 182 discussion groups across 45 intervention clusters began independent dialogue with results from the baseline. This plurality of dialogues, rather than attempting a single concerted community strategy in these highly divided communities, might have been a key to success of the intervention. Between them, the multiple community groups owned the dengue problem and the solutions. Incorporation of different age groups in the brigades increased the proportion of community members they could deal with. The types of community member involved in the pivotal planning discussions varied widely from place to place. Every community opted to engage children, who were good at socialising the evidence among their peers and helpful introducing *brigadistas* to households. The mid-course peer evaluation reinforced this sense of community ownership and achievement we believe was central to impact.

Viewed retrospectively, all community strategies included actions that built an enabling environment for change, providing space for locally authored and locally funded structural changes in the mosquito ecosystem. The repeated house-to-house visits (*visitas de acompañamiento*) to discuss evidence with householders and to inspect household water containers almost certainly set a tone for the overall mobilisation. Although details differed from place to place, all communities chose popular communication activities such as posters and leaflets, graffiti, murals and banners, street theatre, parades, sports events and games (lotteries, brain teasers, and *piñatas*). In the early stages of the intervention these dissemination channels provided new information but, as everyone soon had the same information about the mosquito life cycle, their greater function is likely to have been “social space” -- an enabling environment for household and community action to change the mosquito ecosystem.

*Ecosystem changes* adopted by communities included household and community action to eliminate early stages of the mosquito and, by putting lids on water tanks, to prevent mosquitoes laying eggs. Communities also reintroduced their own biological control using larvivorous fish. We see these locally defined and environmentally neutral actions as the results of the larger SEPA process, not to be mistaken for isolated or prescribed activities.

### Recycling campaigns to raise money for other activities

Following activities specifically about dengue, some communities began to collect and redeem plastic bottles for cash to support communal works. Schools, supported by parents and *brigadista*s, took the lead to finance improvements in the school premises, such as repainting or repairing walls. Sometimes the recycling programme was community-wide, with the collection centre located at the community commissary and the proceeds being used for collective benefit.

### Increased demand for longer term improvement in services

Some *brigadista*s acted as a bridge between community members and public services. Through collective activities like cleaning of streets, drains and ditches, and repairs to infrastructure, communities learned to deal with public service bureaucracies in an organized way. Community members began to demand sanitation services and submitted petitions to the public works department for infrastructure improvements, such as street paving. One community, after successfully obtaining the cooperation of the sanitation services in a street cleaning campaign, arranged monthly clean-ups in collaboration with the local sanitation department, water suppliers, and public works.

### Rise of new leadership

The mobilisation gave rise to new leadership in some communities. *Brigadistas* who were school drop-outs, those who could not read or write very well, and those who had never spoken before in public found voice and confidence in talking to their neighbours about dengue. They gained the respect and confidence of their neighbours as a result of their work. Sharing new knowledge about the life cycle of the mosquito and how to interrupt it motivated them to think of new ways of doing so, calling upon existing talents in the neighbourhood for singing, song-writing, dancing, and visual arts.

### Stimulus to volunteerism and increasing social capital

The first wave of *brigadistas* received a small stipend for their work – enough to cover a meal a day for one person at local market prices. Within a couple of months, especially in communities outside Acapulco and in indigenous communities, increasing numbers of community volunteers joined in the effort without pay, trebling the original number of *brigadistas*. Over time, growing household awareness of the need to control breeding sites outside of individual households led to collective clean-up campaigns in the streets and public spaces like cemeteries.

We are aware that aspects of the intervention impacted negatively on the government vector control programme. Some people removed temephos in order to place fish in their water tanks to control mosquito breeding; although households using fish were at lower risk of dengue [23], it is possible the wish to use fish disrupted the official programme in clusters where fish were widely used. We have no evidence about how the community engagement and organisation might affect a dengue vaccination campaign. Our general sense is that this would be if anything positive, as people are more informed and motivated to increase their protection.

In the exceptionally difficult and dangerous field conditions of Mexico’s Guerrero state, it was community authorship and subsequent ownership of the intervention that had an impact. Reproducing our success elsewhere will require building that authorship and ownership, rather than simply copying the specific preventive activities the communities opted to do.

## Abbreviations

CIET: Centro de Investigación de Enfermedades Tropicales
ISRCTN: International Standard Randomised Controlled Trial Number
SEPA: Socialisation of evidence for participatory action
